# BLOOD BIOMARKERS ASSOCIATED WITH POSTOPERATIVE DELIRIUM INCIDENCE, SEVERITY, DURATION AND SUBTYPE

**DOI:** 10.1093/geroni/igae098.3812

**Published:** 2024-12-31

**Authors:** Hyangkyu Lee, Hyeonmi Cho, Jeongeun Choi, Hyunki Park

**Affiliations:** Yonsei University, Seoul, Republic of Korea; Yonsei University, Seoul, Republic of Korea; Yonsei University, Seoul, Republic of Korea; Yonsei University, Seoul, Republic of Korea

## Abstract

To evaluate the associations of neurological biomarkers —specifically tau, phosphorylated tau, amyloid β40, amyloid β42, amyloid β42/40 ratio, glial fibrillary acid protein (GFAP), neurofilament light (NfL), and brain-derived neurotrophic factor (BDNF) —with the incidence, severity, duration, and subtype of postoperative delirium. In a group of patients over 70 years old who had spine surgery, delirium patients (n=95) and non-delirium patients (n=95) were matched 1:1 with propensity score matching using demographic, lifestyle, and clinical characteristics. Neurological biomarkers were measured after collecting peripheral blood samples immediately after the surgical procedure in the recovery unit. Delirium was observed twice a day from the first to the third postoperative day, and once a day thereafter. Although there were no significant associations between biomarkers and the incidence of postoperative delirium, patients who developed postoperative delirium, each 1 standard deviation increase in NfL was associated with a 1.51 increase in the peak DRS-R-98 severity score (95% confidence interval: 0.07 to 2.95). Each 1 standard deviation rise in GFAP and NfL was associated with an odds ratio of 2.08 (95% confidence interval: 1.15 to 3.74) and 2.35 (95% confidence interval: 1.13 to 4.92), respectively, for delirium duration of 4 days or more. The odds ratio for the hypoactive subtype of delirium increased by 1 standard deviation in A□42 (95% CI: 1.28 to 6.66). Our study demonstrated that blood biomarkers could be useful in predicting the severity, duration, and subtype of postoperative delirium among older adults.

